# Recurrent Pericardial Effusion in a Patient With Delayed Progression of Melanoma Treated With Immune Checkpoint Inhibitors

**DOI:** 10.7759/cureus.47727

**Published:** 2023-10-26

**Authors:** Elsie A Valencia, Natalie Anumolu, Pinky Jha

**Affiliations:** 1 Medicine, Medical College of Wisconsin, Wauwatosa, USA; 2 Internal Medicine, Medical College of Wisconsin, Wauwatosa, USA

**Keywords:** immune checkpoint inhibitors, melanoma, ipilimumab nivolumab, pericardial effusion, pericarditis, immune-related adverse events

## Abstract

Two commonly used immune checkpoint inhibitors (ICIs) utilized in the treatment of metastatic melanoma are nivolumab, a programmed death (PD-1) checkpoint inhibitor, and ipilimumab, a cytotoxic T-lymphocyte antigen (CTLA-4) checkpoint inhibitor. However, due to the activation of the immune system, ICIs have been associated with cardiotoxic immune-related adverse events (irAEs). Here, we present a 40-year-old male with stage 4 metastatic melanoma treated with nivolumab and ipilimumab who developed recurrent pericardial effusions and subsequent constrictive pericarditis 10 months after initiation of treatment. He initially received a total of four cycles and was started on maintenance nivolumab on 8/2022. On 3/23/2023, he complained of chest pain and was found to be hypotensive. He subsequently underwent an emergent pericardiocentesis where 330cc of serosanguinous fluid was drained. Repeat echo on 3/24 demonstrated a re-accumulation of a moderate-sized pericardial effusion, and a subxiphoid pericardial window was placed. He again presented on 5/24/2023 with similar complaints, and a CT scan of chest showed enlarged pericardial effusion with new bilateral pleural effusions.

To our knowledge, this is one of few case reports discussing pericardial effusions in the setting of nivolumab and ipilimumab ICI immunotherapy.

## Introduction

Immune checkpoint inhibitors (ICIs) have revolutionized the treatment of metastatic melanoma since they were approved a decade ago as they allow for a more robust immune response to tumor cells. Two commonly used ICIs used in this setting are nivolumab, a programmed death (PD-1) checkpoint inhibitor, and ipilimumab, a cytotoxic T-lymphocyte antigen (CTLA-4) checkpoint inhibitor. Both function as monoclonal antibodies that prevent T cells from being inhibited, allowing them to multiply and destroy tumor cells [1]. However, due to the activation of the immune system, ICIs have been associated with immune-related adverse events (irAEs). irAEs usually involve the gastrointestinal tract or skin, although they may occasionally affect the cardiovascular system [2]. Cardiotoxic irAEs are uncommon. Consequently, there are not many reports on immune-related pericardial effusions in relation to ICIs [3].

Here, we present a 40-year-old male with stage 4 metastatic melanoma treated with nivolumab and ipilimumab who developed recurrent pericardial effusions and subsequent constrictive pericarditis. This is one of the few case reports discussing pericardial effusions in the setting of nivolumab and ipilimumab ICI immunotherapy.

## Case presentation

A 40-year-old male with a past medical history significant for Hodgkin’s lymphoma s/p chemotherapy and radiation, hypothyroidism, and metastatic melanoma presented to the Emergency Department (ED) in early 2023 with epigastric chest pain and shortness of breath secondary to recurrent pleural effusion. Of note, the patient was started on immunotherapy (ipilimumab and nivolumab) a year ago for his metastatic melanoma with ongoing maintenance therapy.

A review of the patient’s oncologic history revealed the diagnosis of Hodgkin’s lymphoma a decade ago which was treated with ABVD chemotherapy, doxorubicin, and bleomycin. In early 2020, he was found to have extensive metastatic disease with lesions in the brain, skin, bone, muscle, myocardium, lungs, and liver. A recurrence of his Hodgkin's lymphoma was initially considered the source of his metastatic disease; however, upon tissue biopsy, these metastases were confirmed to be melanoma. A palliative regimen of ipilimumab 3 mg/kg and nivolumab 1 mg/kg was started. He received one dose of this treatment and was subsequently found to be BRAF V600E positive for which he was initiated on dabrafenib and trametinib in mid-2020. He had a good response to treatment with improved brain imaging. This continued until early 2022 when he began experiencing fevers to 105F which improved after stopping dabrafenib and trametinib. His disease continued to progress with recurrence of brain metastases, so ipilimumab and nivolumab were restarted in 2022. He received a total of four cycles and was started on maintenance nivolumab in late 2022.

**Figure 1:**
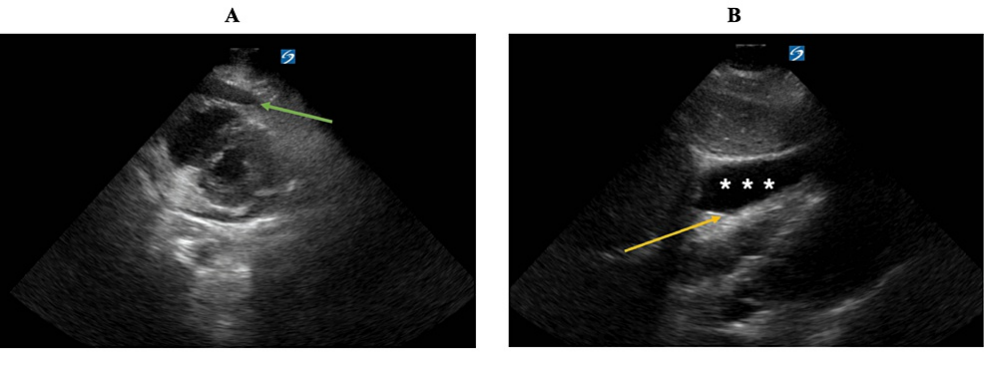
Cardiac ultrasound showcasing pericardial effusion with tamponade physiology A: Cardiac ultrasound with evidence of large pericardial effusion on short axis view (green arrow). B: Cardiac ultrasound with evidence of pericardial effusion (asterisks) and of early diastolic RV collapse consistent with tamponade physiology (yellow arrow) on parasternal long axis view.

He presented to the ED in early 2022 with complaints of chest pain and shortness of breath. A CT scan showed a small pericardial effusion without evidence of tamponade, so he was discharged home. The following day, he returned to the ED with worsening chest pain and hypotension. A cardiac ultrasound revealed a large pericardial effusion with evidence of RV collapse suggestive of cardiac tamponade (Figure 1). He subsequently underwent an emergent pericardiocentesis where 330cc of serosanguinous fluid was drained. A repeat echo was performed 12 hours after drainage which demonstrated a re-accumulation of a moderate-sized, circumferential pericardial effusion measuring up to 12mm. Subsequently, the decision was made to place a subxiphoid pericardial window; tissue obtained during window placement was biopsied. Final pathology from this pericardium tissue showed acute inflammation and fibrinous exudate with no evidence of malignant cells. He was discharged home on day 5. Due to concern for pericarditis in the setting of EKG changes and elevated inflammatory markers, he was discharged on colchicine and non-steroidal anti-inflammatory drugs.

In early 2023, he again presented to the ED with similar symptoms as before including non-radiating epigastric pain, tachycardia, and worsening shortness of breath. His vital signs were as follows: temperature of 98.4, heart rate of 145 beats per minute, blood pressure of 109/67, respiratory rate of 20, and oxygen saturation of 98%. His physical examination revealed a tachycardic rate but was otherwise normal.

**Figure 2:**
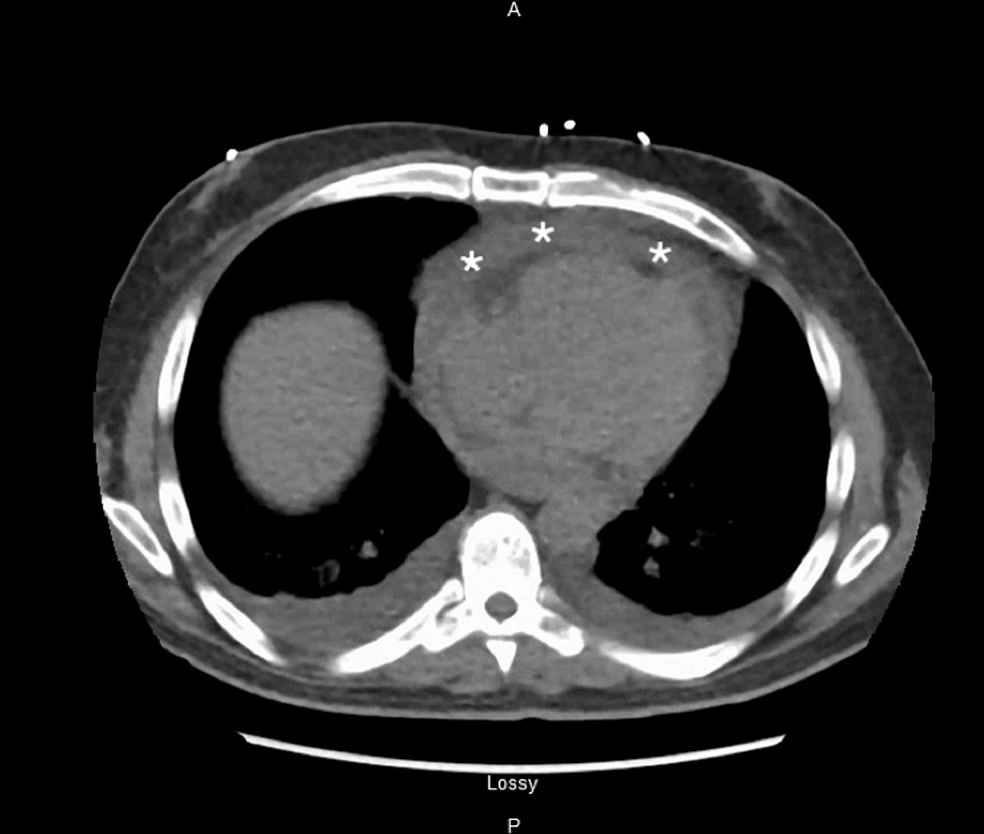
CT scan showing pericardial effusion (asterisks)

His admission laboratory tests were unremarkable except for the presence of elevated markers of ESR and CRP, which were found to be elevated to 38 (<25 mm/hr) and 21.27 (<0.50 mg/dL), respectively. An echocardiogram was performed which revealed a new possible fibrinous material or clot in the pericardial space. A CT scan of his chest showed an enlarged pericardial effusion with new bilateral pleural effusions (Figure 2).

A right and left heart cardiac catheterization was performed with findings suggestive of constrictive pericarditis (Table 1). He was discharged home on day 3 with colchicine 0.6 mg twice daily and ibuprofen 600 mg three times daily. He was instructed to follow up with outpatient cardiology. At his follow-up appointment, his inflammatory markers continued to be elevated with resurgence of pericarditis symptoms, so he was transitioned from ibuprofen to prednisone 40 mg. The patient continues to follow up with the outpatient physician.

**Table 1:** Cardiac catheterization findings RVEDP: Right ventricular end diastolic pressure; RV: Right ventricular; LV: left ventricular; RA: right atrial

Diagnostic Conclusions
Equalization of diastolic pressures
Prominent X & Y descent in RA waveform
Discordance of the RV and LV tracings with respirations; square root sign within the LV pressure tracing
Increased RVEDP relative to RV systolic pressure

## Discussion

Here, we report a rare case of recurrent pericardial effusions and subsequent constrictive pericarditis in the context of ICI immunotherapy for metastatic melanoma. ICIs have been revolutionary in the treatment of metastatic melanoma. Nivolumab and ipilimumab are immunomodulators commonly used in this setting, preventing T cells from being inhibited. This allows T cells to multiply and destroy malignant cells [1]. However, due to the activation of the immune system, ICIs have been associated with irAEs which typically affect the gastrointestinal tract or skin [2]. irAEs affect the cardiovascular system approximately 1% of the time, making them a rare but life-threatening occurrence [2,3]. Some manifestations of cardiotoxicity include cardiac arrest, myocarditis, heart failure, and pericardial disease [3].

The pathophysiology of cardiotoxic irAEs is not entirely understood. One proposed mechanism suggests that ICIs inhibit interactions between PD-1 and programmed cell death ligand 1 (PDL-1) leading to the activation of T-lymphocytes. These T cells destroy malignant cells; however, they may also target healthy cardiac tissue. Another proposed mechanism suggests that molecular mimicry induces cardiac tissue autoimmunity [4]. Cardiotoxic irAEs presenting as pericardial disease are uncommon, representing approximately 0.36% of reported toxicities [5].

In our literature review of cardiotoxic irAEs, we found 10 prior cases of pericardial effusions (Table 2). To our knowledge, this is the 11th case report discussing pericardial effusions in the setting of nivolumab and ipilimumab immunotherapy.

**Table 2:** Prior cases of nivolumab and/or ipilimumab-related pericardial effusions

Author	Journal	Year	Sex	Age	ICI type
Yun et al. [6]	Case Rep Oncol Med	2015	Male	59	Ipilimumab (CTLA-4)
Nesfeder et al. [7]	Int J Cardiol	2016	Male	64	Nivolumab (PD-1)
Kushnir et al. [8]	Cardiology	2017	Male	67	Nivolumab (PD-1)
de Almeida et al. [9]	J Immunother	2018	Male	69	Nivolumab (PD-1)
Naime et al. [10]	J Cancer Ther	2018	Male	52	Nivolumab (PD-1)
Shaheen et al. [11]	Exp Hematol Oncol.	2018	Female	70	Nivolumab (PD-1)
Altan et al. [12]	J Thorac Oncol	2019	Female	65	Nivolumab (PD-1) & Ipilimumab (CTLA-4)
Saade et al. [13]	J Immunother Cancer	2019	Female	58	Nivolumab (PD-1)
Saade et al. [13]	J Immunother Cancer	2019	Male	65	Nivolumab (PD-1)
Saade et al. [13]	J Immunother Cancer	2019	Female	55	Nivolumab (PD-1)

Cases listed in Table 2 were determined to be irAEs rather than pseudo-progression. Pseudo-progression is characterized by a temporary increase in the tumor size followed by regression or the appearance of new lesions. At the time of diagnosis, distinguishing between irAEs and pseudo-progression is a difficult task; ICI therapy is known for causing pseudo-progression [14]. Therefore, pericardial effusions may sometimes be due to this phenomenon rather than a true irAE. irAEs are typically a diagnosis of exclusion [15]. Differentiating between the two is crucial as diagnosis will dictate treatment: pseudo-progression typically resolves spontaneously or with continued ICI therapy, whereas irAEs often require the discontinuation of ICIs and the initiation of immunosuppressive treatment, such as corticosteroids [16]. This can be done using imaging, biopsy, or fluid analysis [17]. In the case of our patient, pericardial effusion was determined to be immune-related due to tissue biopsy results as well as resolution of symptoms upon discontinuation of ICI therapy.

Due to the rare occurrence of pericardial toxicity, there is limited data on outcomes; however, prior literature has found that they typically have a poor prognosis with a reported mortality rate of anywhere from 13 to 21% [3]. The timeframe in which cardiotoxicity occurs after the initiation of ICIs is variable. In this case report, pericardial disease occurred 10 months after initiation of treatment. Based on prior research, the majority of toxicities occur within one year; however, reports have shown that they can also occur years after initiation of ICIs [3]. Due to this, several studies recommend performing routine cardiac surveillance in patients receiving ICIs, including a baseline cardiovascular assessment [3,4].

## Conclusions

We present this case report which highlights the rarity and severity of cardiotoxic irAEs associated with ICI immunotherapy, specifically nivolumab and ipilimumab, in the context of metastatic melanoma treatment. With a reported mortality rate of up to 21%, pericardial effusions and subsequent constrictive pericarditis, though infrequent, underscore the importance of vigilance in monitoring cardiovascular health during and after ICI therapy. The mechanisms behind cardiotoxic irAEs remain incompletely understood, emphasizing the need for further research to elucidate their pathophysiology and identify strategies to minimize their risk. 
